# Radiofrequency Ablation Versus Laser Neurolysis of the Posterior Nasal Nerve in Patients With Chronic Rhinitis

**DOI:** 10.1002/ohn.70156

**Published:** 2026-02-06

**Authors:** Chia‐Chen Lin, Yi‐Li Hwang, Jyun‐Yi Liao, Han‐Lo Teng, Ting‐Yu Shih, Chien‐Yu Huang

**Affiliations:** ^1^ Department of Otorhinolaryngology & Head and Neck Surgery New Taipei Municipal TuCheng Hospital (Built and Operated by Chang Gung Medical Foundation) New Taipei City Taiwan, ROC; ^2^ Department of Oral and Maxillofacial Surgery Chia‐Yi Chang Gung Memorial Hospital Chia‐Yi County Taiwan, ROC; ^3^ Department of Otolaryngology‐Head and Neck Surgery Ditmanson Medical Foundation Chia‐Yi Christian Hospital Taiwan, ROC; ^4^ Department of Otolaryngology, Chiayi Hospital Ministry of Health and Welfare Taiwan; ^5^ Department College of Artificial Intelligence National Yang‐Ming Chiao Tung University Taiwan, ROC

**Keywords:** allergy, posterior nasal nerve neurolysis, radiofrequency, rhinitis

## Abstract

**Objective:**

To compare the clinical outcomes of radiofrequency ablation of the intraturbinate segment of the posterior nasal nerve (RAPN) alone versus combined RAPN with CO₂ laser posterior nasal nerve neurolysis (RPN3) in patients with chronic rhinitis refractory to medical therapy.

**Study Design:**

Retrospective cohort study.

**Setting:**

Single tertiary care center.

**Methods:**

Adult patients with chronic rhinitis unresponsive to medical treatment for over 6 months who underwent either RAPN or RPN3 between February 2023 and May 2024 were included. Inclusion criteria were 24‐hour reflective total nasal symptom score (rTNSS) ≥ 3, rhinorrhea score ≥1, and NOSE score ≥11. Exclusion criteria included chronic rhinosinusitis, nasal polyps, and coagulopathy. Demographics and comorbidities were documented. Primary endpoints were changes in rTNSS, NOSE scores, and response rate (≥30% improvement from baseline) at 1‐, 3‐, and 6‐months posttreatment.

**Results:**

A total of 101 patients were analyzed (RPN3: 76; RAPN: 25). Baseline rTNSS and NOSE scores were comparable between the two groups (*P* = .144 and .414, respectively). Both groups demonstrated significant improvements in rTNSS and NOSE scores at all follow‐up points (*P* < .001). From 1 to 6 months, RPN3 achieved significantly greater improvements compared to RAPN (*P* = .039‐.045), particularly in the rhinorrhea and nasal itching sub‐scores (*P* = .003‐.039). Response rates for rTNSS ranged from 91% to 100% in the RPN3 group and 84% to 96% in the RAPN group.

**Conclusion:**

Both RAPN and RPN3 are effective in treating chronic rhinitis unresponsive to medication. The addition of CO₂ laser posterior nasal nerve neurolysis (RPN3) provides superior symptomatic relief, particularly for rhinorrhea and nasal itching.

Chronic rhinitis is a prevalent inflammatory disorder of the nasal mucosa, characterized by persistent symptoms such as congestion, rhinorrhea, sneezing, and itching, which can significantly impair patients' quality of life.^1^ For individuals who remain symptomatic despite prolonged medical therapy, posterior nasal nerve neurolysis (PNNN) has emerged as a viable interventional strategy,^2^ achievable through surgical dissection, cryotherapy, or radiofrequency (RF) ablation.^3^ Current guidelines from the American Academy of Otolaryngology support PNNN in medically refractory cases.^4^

Radiofrequency‐based PNNN has shown favorable long‐term outcomes,^5^, ^6^, ^7^, ^8^ but device limitations and regulatory barriers have driven the exploration of alternative techniques such as diode laser neurolysis^9^ and radiofrequency ablation of the posterior turbinate segment (RAPN).^10^

We previously introduced a novel combined approach—RAPN followed by CO₂ laser PNNN—termed RPN3, which demonstrated superior short‐term efficacy compared to RF ablation alone.^11^ Subsequent studies reported high response rates and significant improvements in both Reflective Total Nasal Symptom Score (rTNSS) and Nasal Obstruction Symptom Evaluation (NOSE) scores, suggesting enhanced outcomes through dual mechanisms: RAPN reduces turbinate volume, while CO₂ laser PNNN provides targeted posterior nerve ablation.^12^ However, the independent contribution of laser PNNN remains unclear due to the lack of direct comparison with RAPN alone. Additionally, prior studies primarily focused on total symptom scores without analyzing individual symptom domains.

To address these gaps, we conducted a retrospective cohort study comparing the clinical efficacy of RAPN alone versus combined RPN3 in patients with chronic rhinitis unresponsive to medical therapy. We extended the follow‐up to six months and analyzed both overall and symptom‐specific outcomes to evaluate the added benefit and durability of laser PNNN. By elucidating the distinct therapeutic contributions of each intervention, this study aims to inform procedural decision‐making and optimize treatment strategies for chronic rhinitis.

## Methods

### Study Design and Patient Population

This retrospective cohort study evaluated patients who sought treatment for chronic rhinitis between February 2023 and May 2024. The study protocol was approved by the Ethics Committee of Chia‐yi Christian Hospital (Approval No: CYCH2024022; April 12, 2024). Patients were screened using questionnaires and nasal endoscopy to exclude sinusitis and other nasal pathologies that could confound the results. Inclusion criteria were as follows: age between 18 and 65 years, chronic rhinitis refractory to medical therapy for more than 6 months (defined as persistent symptoms despite the use of intranasal corticosteroid sprays and oral antihistamines), a reflective total nasal symptom score (rTNSS) ≥3, a rhinorrhea subscore ≥1, and a nasal obstruction symptom evaluation (NOSE) score ≥11. Exclusion criteria included the presence of acute or chronic rhinosinusitis, coagulopathy, concurrent surgeries involving the oral cavity, and anatomical obstructions that limited access to the posterior nasal cavity in patients who declined corrective procedures (e.g., septoplasty). Baseline rhinitis medication use was recorded in Table 1 to assess potential confounding; medications were not restricted before or after surgery.

**Table 1:** Patient Demographics, Baseline Characteristics, and Procedural Information

	RAPN	RPN3	
	N = 25	N = 76	*P* value
Male sex	19 (76)	45 (59.2)	.133
Age (years)	36 ± 11.1	36.9 ± 7.8	.668
BMI, kg/m^2^	25.6 ± 5.7	25.5 ± 3.8	.954
OSA	8 (32)	8 (10.5)	.01*
Rhinitis medicantosa	2 (8)	15 (19.7)	.177
Previous nasal surgery	2 (8)	10 (13.2)	.494
Lidocaine 2%, mean ± sD	5.3 ± 0.3	6.3 ± 0.3	<.001
Anethesia			
Local	22 (78)	64 (84.2)	.648
General	3 (12)	12 (15.8)	
Septoplasty	3 (12)	3 (7.5)	.839
Adenoidectomy	2 (8)	2 (2.6)	.237
Baseline rTNSS	7.4 ± 2.5	8.2 ± 2.3	.144
Obstruction	2.6 ± 0.5	2.6 ± 0.6	.951
Rhinorrhea	1.7 ± 0.8	2.1 ± 0.8	.069
Itching	1.4 ± 0.8	1.8 ± 0.9	.108
Sneezing	1.6 ± 1	1.7 ± 0.9	.495
Baseline NOSE	13.8 ± 2.2	14.3 ± 2.8	.414
Obstruction	3.2 ± 0.6	3.1 ± 0.8	.685
Stuffiness	2.4 ± 1.0	2.8 ± 0.9	.091
Trouble breathing	2.9 ± 0.7	3.0 ± 0.8	.698
Trouble sleep	2.4 ± 1.1	2.8 ± 0.8	.092
Trouble exercise	2.8 ± 0.7	2.6.2 ± 1.0	.25
Medication			
Intranasal corticosteroids	9 (36)	27 (35.5)	.966
Decongestants	4 (16)	13 (17.1)	.899
Oral antihistamines	10 (40)	25 (32.9)	.522
Intranasal anticholinergic	0 (0)	0 (0)	

Data are presented as mean ± standard deviation or n (%).

Abbreviations: NOSE, nasal obstruction symptom evaluation; OSA, obstructive sleep apnea; RAPN, radiofrequency ablation of the intraturbinate segment of the posterior nasal nerve; RPN3, radiofrequency ablation of the intraturbinate segment of the posterior nasal nerve with laser posterior nasal nerve neurolysis; rTNSS, reflective Total Nasal Symptom Score.

*Statistically significant with *P* < .05.

### Procedure and Treatment Allocation

**Figure 1:**
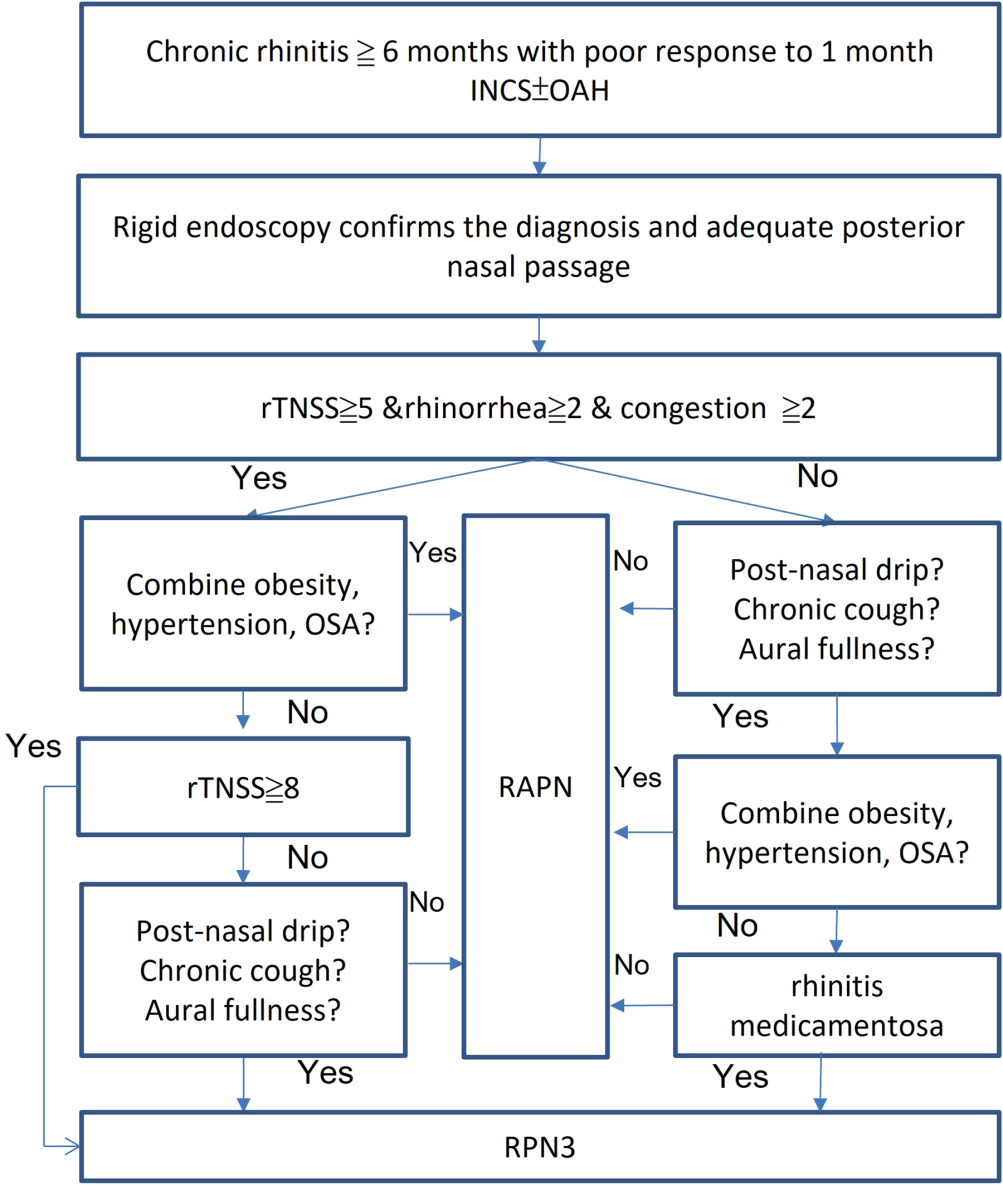
Evidence‐based shared decision‐making algorithms. Flowchart summarizing how patients with chronic rhinitis selected different minimally invasive surgical options.

Surgical options were discussed with each patient and determined through shared decision‐making, considering the patient's history, preoperative rhinitis questionnaire scores, endoscopic findings, and comorbidities. Evidence‐based shared decision‐making algorithms (Figure 1) were applied for discussion. Huang et al^11^ reported an enhanced surgical outcome when adding laser PNNN to RF alone in patients with TNSS ≧5, rhinorrhea ≧2, and congestion ≧2. When patients were comorbid with hypertension, obesity, and OSA, post‐operation complication risk was significantly higher than in the usual group, so less invasive procedures were preferred.^13^ Stolovitzky et al and Lee et al reported up to 3‐year improvement of postnasal drip and cough symptoms after the PNNN technique.^5^, ^14^ Yen et al had published 2‐year follow‐up results for improved ETDQ‐7 and auricular fullness symptoms after the PNNN procedure.^15^ Thus, when the patient complains of these symptoms, RPN3 may provide additional control. Liao et al reported that combining laser posterior nasal nerve treatment with RF turbinates alone may offer additional benefits by providing enhanced control of nasal itching and achieving an earlier response in rhinorrhea and sneezing control.^16^

The RPN3 approach was explained to patients as involving a longer surgery time (approximately 30 vs 20 minutes), the use of more local anesthesia, and potentially greater procedural discomfort in the posterior nasal cavity. Patients were also informed of the potential increased risk of complications, such as posterior epistaxis. They were performed as day surgery under local anesthesia when conducted alone, and under general anesthesia when combined with septoplasty or adenoidectomy. All procedures were performed in the operating room. Approximately 2 mL of 2% lidocaine without epinephrine was injected into the anterior pole of both inferior turbinates. In the RPN3 group, additional lidocaine was injected at the junction of the middle turbinate and the inferior insertion into the lateral nasal wall to infiltrate the area of distribution of the posterior nasal nerve. On average, approximately 1 mL more lidocaine was used compared to the RAPN group.

RAPN was performed using Olympus Celon Elite ESG‐200 (Olympus Europa). A single‐use disposable stylus delivered bipolar RF energy at 15 W to the inferior turbinates. The RF was applied to 15 to 30 nonoverlapping positions on the inferior turbinate to reduce the turbinate size and also to ablate the intraturbinate neurovascular structures at the posterior part of the inferior turbinate. The procedure took approximately 20 minutes for both sides.

In the RPN3 group, after the completion of bilateral RAPN, PNNN was performed using a 2 W continuous wave laser with AcuPulse^TM^ CO_2_ laser (Lumenis Ltd.) that was transmitted via a nasal probe angulated at 20° and a straight‐tip 90° mirror, with smoke evacuation. The posterior middle meatus and a super‐posterior portion of the inferior turbinate were cauterized to target the region supplied by the posterior nasal nerve. This procedure took approximately 5 min/side.

After the procedure, the inferior turbinate was covered with Hemopatch (Baxter International), a thin sealing hemostat. A compression tamponade or nasal irrigation was not required after the surgery. Antihistamine, antibiotics, and analgesic were prescribed for 3 days.

### Follow‐Up Interventions

Patients were followed at 1, 3, and 6 months after surgery. At each visit, standardized questionnaires were administered to assess clinical outcomes, complications, and postoperative discomfort. Patients were carefully evaluated, and local treatments (eg, suction of nasal discharge or crust from the middle meatus) were provided when necessary. Nasal endoscopy was performed in patients reporting discomfort, including persistent rhinorrhea, nasal itching, or suspected infection. Rhinitis medications were not prescribed routinely but were provided only upon patient request, typically as short‐term prescriptions for severe rhinorrhea or itching.

### Surveys

Patients were evaluated using a 24‐hour rTNSS, Nasal Obstruction Symptom Evaluation (NOSE) scale.^17^, ^18^ The 24 hour rTNSS is a 4‐item questionnaire with a range of 0 to 20. The questionnaire focuses on the patient's recent symptoms of nasal congestion, rhinorrhea, itching, and sneezing within 24 hours. The NOSE scale is a nasal symptom‐related quality of life questionnaire composed of 5 items scored between 0 and 100, and it reflects the quality of life restrictions in patients with nasal problems.^18^

### Outcome Evaluation

The primary endpoint was the interval change of rTNSS and NOSE questionnaire scores and a score of each item in the scoring systems (subclass score) through follow‐up visits. The secondary endpoint was an improvement of ≥30% in the 24 hours rTNSS compared with the baseline score. Side effects such as rhinorrhea, pain, crusting, and sneezing within 1 week postoperatively were documented. Complications such as epistaxis, dry eyes, and headaches were also documented.

### Statistics

Continuous data are presented as means and standard deviation in the tables and 95% confidence intervals (CIs) in the figures. Categorical data are presented as counts (frequencies) and percentages unless otherwise specified. Changes in the survey scores from baseline were analyzed using two‐tailed paired *t*‐tests for normally distributed data. The Wilcoxon signed‐rank test was used to analyze data that did not meet normal distribution assumptions. Statistical analyses were performed using PASW Statistics (version 18.0.0; SPSS Inc.).

## Results

**Figure 2:**
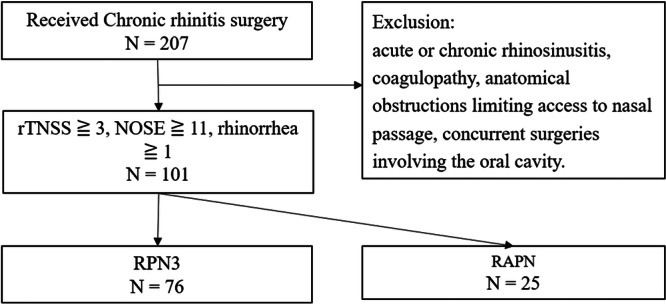
Patient disposition. 101 patients included: 76 received RPN3 and 25 received RAPN. rTNSS, 24‐h reflective total nasal symptom score.

A total of 101 patients underwent surgical treatment for resistant chronic rhinitis, with 76 receiving RPN3 and 25 receiving RAPN (Figure 2). There were no significant differences between the groups regarding age, sex, BMI, prevalence of rhinitis medicamentosa, or history of nasal surgeries. However, the prevalence of obstructive sleep apnea (OSA) and the use of lidocaine as local anesthesia were significantly higher in the RPN3 group compared to the RAPN group (32% vs 10.5%, *P* = .01; 6.3 ± 0.3 vs 5.3 ± 0.3, *P* < .001). The greater amount of lidocaine used in the RPN3 group was due to the inclusion of an additional procedure, PNNN, which was not performed in the RAPN group. Baseline rTNSS was slightly higher in the RPN3 group than in the RAPN group (8.2 ± 2.3 vs 7.4 ± 2.5; *P* = .144), although the difference was not statistically significant. Similarly, baseline NOSE scores were comparable between groups (14.3 ± 2.8 for RPN3 vs 13.8 ± 2.2 for RAPN; *P* = .414). Baseline subscores for all four rTNSS domains were consistently higher in the RPN3 group, indicating a trend toward greater symptom burden, although the differences were not statistically significant (Table 1).

**Figure 3:**
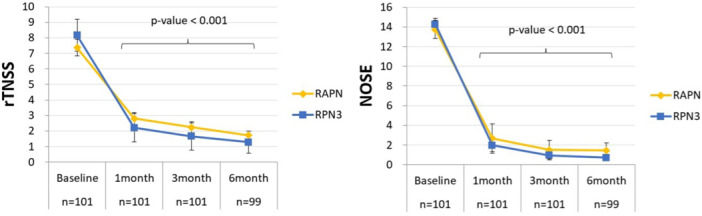
rTNSS and NOSE score changes over time. Both groups improved significantly at each follow‐up (*P* < .001). RAPN, RPN3 as defined above.

As shown in Table 2 and Figure 3, the RPN3 group demonstrated significant improvements in rTNSS at 1, 3, and 6 months postoperatively compared to baseline (*P* < .001 at each time point). Moreover, RPN3 achieved significantly greater rTNSS improvement than RAPN across the 1‐to 6‐month follow‐up period (*P* = .032‐.045). Although NOSE scores also showed numerical improvement in both groups, the differences between groups were not statistically significant across the follow‐up period (*P* = .089‐.191).

**Table 2:** Changes in rTNSS and NOSE Scores During Follow‐Up

	BL	95% CI	1 month	95% CI	3 month	95% CI	6 month	95% CI
rTNSS								
RAPN	7.4	1	2.8	0.9	2.2	0.9	1.7	0.7
Change			−4.6	1.2	−5.1	1.1	−5.6	1.2
RPN3	8.2	0.5	2.2	0.4	1.7	0.3	1.3	0.3
change			−5.9	0.7	−6.5	0.6	−6.9	0.55
*P*‐value*			.045		.032		.039	
NOSE								
RAPN	13.8	0.9	2.7	1.5	1.5	1	1.4	0.8
Change			−11	1.9	−12.3	1.4	−12.3	1.4
RPN3	14.3	0.6	2	0.6	0.9	0.3	0.7	0.3
Change			−12.3	0.9	−13.3	0.7	−13.6	0.7
*P*‐value*			.191		.139		.089	

Mean scores and 95% confidence intervals (CI) are presented at baseline, 1‐, 3‐, and 6‐month follow‐up.

Abbreviation: CI, confidence interval.

* *P*‐values indicate intergroup comparisons at each follow‐up time point.

In Table 3 and Supplemental Figure, available online, subscore analysis revealed that the RPN3 group achieved significantly greater improvements in rhinorrhea and nasal itching compared to the RAPN group (*P* = .003‐.039). While improvements in nasal obstruction and sneezing subscores were observed in both groups, these differences did not reach statistical significance.

**Table 3:** Subscore Analysis of rTNSS Changes During Follow‐Up

	BL	95% CI	1 month	95% CI	3 month	95% CI	6 month	95% CI
Obstruction								
RAPN	2.6	0.2	0.6	0.3	0.3	0.2	0.4	0.2
Change			−2	0.4	−2.3	0.3	−2.3	0.3
RPN3	2.6	0.1	0.5	0.1	0.3	0.1	0.2	0.1
Change			−2.2	0.2	−2.4	0.2	−2.4	0.2
*P*‐value*			.408		.743		.452	
Rhinorrhea								
RAPN	1.7	0.3	0.9	0.3	1	0.4	0.6	0.3
Change			−0.8	0.3	−0.8	0.3	−1.1	0.4
RPN3	2.1	0.2	0.8	0.1	0.6	0.1	0.5	0.1
Change			−1.3	0.2	−1.4	0.2	−1.6	0.2
*P*‐value*			.026		.003		.027	
			
Itching								
RAPN	1.4	0.3	0.5	0.1	0.5	0.3	0.4	0.2
Change			−0.9	0.4	−1	0.4	−1.1	0.4
RPN3	1.8	0.2	0.4	0.2	0.3	0.1	0.2	0.1
Change			−1.3	0.2	−1.4	0.2	−1.5	0.2
*P*‐value*			.09		.038		.039	
			
Sneezing								
RAPN	1.6	0.4	0.7	0.3	0.5	0.3	0.4	0.2
Change			−0.8	0.4	−1.1	0.4	−1.2	0.5
RPN3	1.7	0.2	0.6	0.1	0.5	0.1	0.3	0.1
Change			−1.1	0.3	−1.3	0.2	−1.4	0.2
*P*‐value*			.226		.469		.334	

Mean subscores for nasal obstruction, rhinorrhea, itching, and sneezing are presented along with 95% CI.

Abbreviations: CI, confidence interval; RAPN, radiofrequency ablation of the intraturbinate segment of the posterior nasal nerve; RPN3, radiofrequency ablation of the intraturbinate segment of the posterior nasal nerve with laser posterior nasal nerve neurolysis; rTNSS, reflective total nasal symptom score.

* *P*‐values indicate intergroup comparisons at each follow‐up time point.

**Figure 4:**
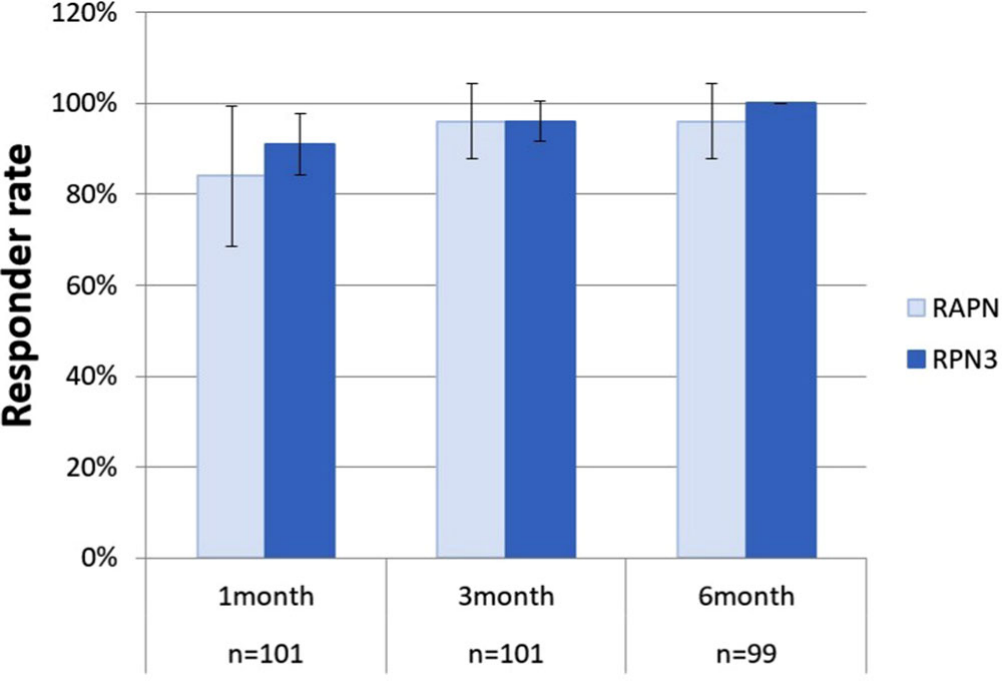
Responder rates. RPN3 (N = 76) achieved 100% response at 6 months versus 96% for RAPN (N = 25). RPN3 outperformed RAPN at all follow‐ups. RAPN, RPN3, rTNSS as defined above.

The rTNSS responder rates—defined as a ≥ 30% reduction from baseline—ranged from 91% to 100% in the RPN3 group and from 84% to 96% in the RAPN group over the 6‐month follow‐up (Figure 4). Although the responder rates in the RPN3 group were slightly higher at 1 month (91% vs 84%), 3 months (96.1% vs 96%), and 6 months (100% vs 96%), these differences were not statistically significant (*P* = .349, .991, and .327, respectively).

Complications were observed in six patients. In the RPN3 group, 2 cases of anterior epistaxis occurred on postoperative Days 20 and 23 at the left inferior turbinate; both resolved after 3 days of compression without sequelae. Additional complications included one case of sinusitis that resolved after mucopus drainage and a 1‐week course of antibiotics, one wound infection, and one case of prolonged crusting lasting more than 1 month, which was resolved after additional follow‐up visits and complete crust removal by 3 months. In the RAPN group, 1 patient developed sinusitis. All complications were classified as mild to moderate, and no severe adverse events occurred.

During the 3‐month follow‐up, 19 patients underwent endoscopy because of previously suspected complications at 1 month or persistent symptoms: 2 for epistaxis, 2 for suspected sinusitis, 2 for suspected wound infection, 8 for moderate‐to‐severe rhinorrhea, and 5 for persistent nasal itching. By the 3‐month visit, all complications suspected at 1 month had completely resolved. At the 6‐month follow‐up, 6 patients required endoscopy, including 4 for persistent rhinorrhea and 2 for moderate‐to‐severe itching.

During the 1‐month follow‐up, rhinitis medications (eg, intranasal corticosteroids, oral antihistamines) were prescribed only upon request for severe rhinorrhea or itching. A total of three patients received short‐term medication (Fexofenadine HCl 60 mg BID): 2 in the RPN3 group and 1 in the RAPN group, with no significant between‐group difference. At the 3‐ and 6‐month visits, medications were prescribed only for patients reporting moderate‐to‐severe rhinorrhea.

In the RAPN group, three patients (12%) experienced worsening rhinorrhea at 3 months, and 1 patient (4%) showed persistent worsening at 6 months. In the RPN3 group, 1 patient (1.3%) demonstrated worsening rhinorrhea at 3 months but showed substantial improvement by the 6‐month follow‐up. All four patients who experienced worsening rhinorrhea were treated with intranasal corticosteroids and oral antihistamines.

Two patients (one in each group) were lost to follow‐up due to inability to attend scheduled visits or unrelated medical issues. No dropouts were attributable to complications or disease progression.

Overall, RPN3 demonstrated greater improvements in rhinitis symptom control compared to RAPN, with significant reductions in rhinorrhea and nasal itching throughout the 6‐month follow‐up period.

## Discussion

This study demonstrates that combining radiofrequency ablation of the posterior turbinate with adjunctive CO₂ laser posterior nasal nerve neurolysis (RPN3) offers superior symptom control compared to radiofrequency ablation alone (RAPN) in patients with medically refractory chronic rhinitis. Notably, significant improvements were observed in rhinorrhea and nasal itching—two of the most bothersome symptoms in allergic rhinitis.^19^ The responder rate reached 100% in the RPN3 group, indicating a consistent and robust therapeutic effect.

International guidelines, including the 2023 ICAR consensus, endorse posterior nasal nerve (PNN) modulation for patients unresponsive to pharmacologic treatment.^2^ While turbinate surgery alone is known to alleviate symptoms of allergic rhinitis,^20^ our findings reinforce this recommendation and suggest that incorporating PNN neurolysis may further enhance clinical outcomes.^11^, ^12^ Although reducing inferior turbinate hypertrophy is effective for relieving nasal obstruction, it does not adequately address parasympathetically mediated symptoms such as rhinorrhea and nasal itching.^21^ Our results highlight the importance of targeting the posterior nasal nerve for comprehensive symptom control. This finding is further supported by a recent meta‐analysis by Ripp et al, which reported that PNN ablation outperformed turbinate surgeries in improving nasal itching and postnasal drip.^22^ These results align with our own data, reinforcing the selective benefit of posterior nasal nerve targeting these symptom domains.

This study also aligns with a growing body of literature supporting the long‐term efficacy of PNN modulation. For example, a recent 3‐year study using temperature‐controlled radiofrequency (TCRF) for PNN ablation reported a 57.3% reduction in rTNSS and a high responder rate of 79.7%.^5^ Other reviews have shown that PNN ablation typically results in an average 3 to 4‐point decrease in rTNSS, translating to approximately a 50% improvement in symptoms.^23^ In our study, the RPN3 group achieved a 6.9‐point reduction in rTNSS, compared to 5.6 points in the RAPN group—representing a roughly 23% greater improvement. Although our current follow‐up period is limited to 6 months, the sustained and even progressive improvement observed in the RPN3 group suggests the potential for durable symptom relief, warranting longer‐term investigation.

Our use of the CO₂ laser for PNN neurolysis represents an innovative application of a well‐established surgical tool. Widely used in sinonasal surgery due to its precision and favorable safety profile,^24^ the CO₂ laser was selected for its ability to deliver controlled thermal energy to specific neural targets while minimizing collateral tissue injury. By applying the laser to the posterior middle meatus and the superior posterior segment of the inferior turbinate, we effectively targeted the sphenopalatine nerve branches responsible for parasympathetic‐mediated glandular secretion and nasal hyperreactivity.^25^, ^26^ The superior efficacy of RPN3 may result from a more extensive denervation of parasympathetic fibers along the posterior nasal nerve, leading to more effective disruption of reflex pathways associated with rhinorrhea and nasal pruritus.^27^ Although the between‐group differences were modest and may not be clinically meaningful on average, patients in the RPN3 group—who generally had more severe baseline symptoms—showed a marked reduction in severe rhinorrhea and nasal itching during the 3‐ to 6‐month follow‐up. As reflected in Figure 3 by a downward shift in symptom severity distribution, the resolution of moderate‐to‐severe symptoms in some patients may represent meaningful improvement.

The CO₂ laser was applied in a non‐contact fashion, enabling clear visualization of the mucosa and precise avoidance of vascular structures. Notably, no cases of significant postoperative bleeding occurred in either group, despite previous studies reporting severe epistaxis in up to 2% of PNN ablation procedures.^28^ The excellent safety profile observed in our cohort may be attributed to the controlled thermal delivery of our non‐contact technique, which minimizes mucosal trauma and maintains a safe distance from pulsatile vessels. While these results are encouraging, larger studies are warranted to determine whether this method offers a definitive advantage in reducing hemorrhagic risk compared to other modalities.

Clinically, RPN3 is minimally invasive, well‐tolerated, and suitable for outpatient practice. Unlike traditional vidian neurectomy, which carries risks such as dry eyes and palatal numbness, RPN3 provides comparable symptom relief through localized neural modulation confined to the nasal cavity.^29^, ^30^ In our study, no cases of dry eyes or palatal numbness were observed in either group, consistent with the more distal site of nerve injury compared to vidian neurectomy. As summarized in Table 4, the overall complication rates were low and comparable between groups, with only minor events such as anterior epistaxis, prolonged crusting and wound infection, all resolving with conservative management. In addition to delivering substantial symptom relief, particularly for rhinorrhea and nasal itching, RPN3 may also reduce reliance on long‐term medication and lower overall healthcare utilization, making it a safe and cost‐effective alternative to more invasive surgical options.^3^, ^31^

**Table 4:** Adverse Events Recorded During Follow‐Up Visits and Those Required Medical Assistance After the Procedure

	RAPN	RPN3	
	N = 25	N = 76	*P* value
All complications	1 (4)	5 (6.6)	.64
Anterior epistaxis^a^	0	2 (2.6)	.418
Posterior epistaxis	0	0	
Prolonged crusting	0	1 (1.3)	.569
Sinusitis	1 (4)	1 (1.3)	.408
Wound infection	0	1 (1.3)	.569
Dried eye	0	0	
Palate numbness	0	0	

^a^ Anterior epistaxis was bleeding in the anterior part of the turbinates, requiring packing in the emergency department or clinics.

The retrospective nature of this study and the relatively small sample size, particularly in the RAPN group, may limit the generalizability of the results. Nevertheless, our study benefits from consistent baseline characteristics and multi‐timepoint assessments, strengthening the reliability of these preliminary findings. Future prospective, randomized trials with larger cohorts and longer follow‐up will be essential to confirm the durability of RPN3, refine patient selection criteria, and explore additional applications of this dual‐modality approach in chronic rhinitis.

## Conclusion

Radiofrequency ablation with posterior nasal nerve neurolysis (RPN3) appears to be a promising and well‐tolerated treatment modality for patients with resistant chronic rhinitis. Compared to conventional intraturbinate ablation (RAPN), RPN3 provides superior symptom control, particularly in reducing rhinorrhea and nasal itching—symptoms that are often refractory to medical therapy. The continuous improvement observed over the 6‐month follow‐up period suggests the potential for durable efficacy. These findings support the integration of RPN3 into clinical practice as a minimally invasive intervention, especially for patients who fail to respond to standard pharmacological treatments. Future multicenter, prospective randomized studies are needed to confirm these results, refine patient selection criteria, and establish long‐term clinical protocols.

## Author Contributions

**Chia‐Chen Lin**, data collection, manuscript drafting and preparation, critical revision, final approval; **Yi‐Li Hwang**, study design and coordination, critical revision, final approval; **Jyun‐Yi Liao**, participant recruitment, data collection, manuscript preparation, critical revision, final approval; **Han‐Lo Teng**, data collection, manuscript preparation, final approval; **Ting‐Yu Shih**, participant recruitment, final approval; **Chien‐Yu Huang**, study design and coordination, participant recruitment, data collection, manuscript preparation, critical revision, final approval.

## Disclosures

### Competing interests

None.

### Funding source

The study was supported by the Ditmanson Medical Foundation Chia‐Yi Christian Hospital Research Program (R113‐056).

## Supporting information


**Supplemental Figure. Symptom severity distribution.**Severity of rTNSS subscores tracked over time. RPN3 showed greater reductions in rhinorrhea and itching.*RAPN, RPN3 as defined above*.
