# Supplementary figures and images for: Radiofrequency Ablation Versus Laser Neurolysis of the Posterior Nasal Nerve in Patients With Chronic Rhinitis
Source: Otolaryngol Head Neck Surg. 2026 Feb 6;174(4):954–62. doi: 10.1002/ohn.70156 (PMC13035011; doi:10.1002/ohn.70156)

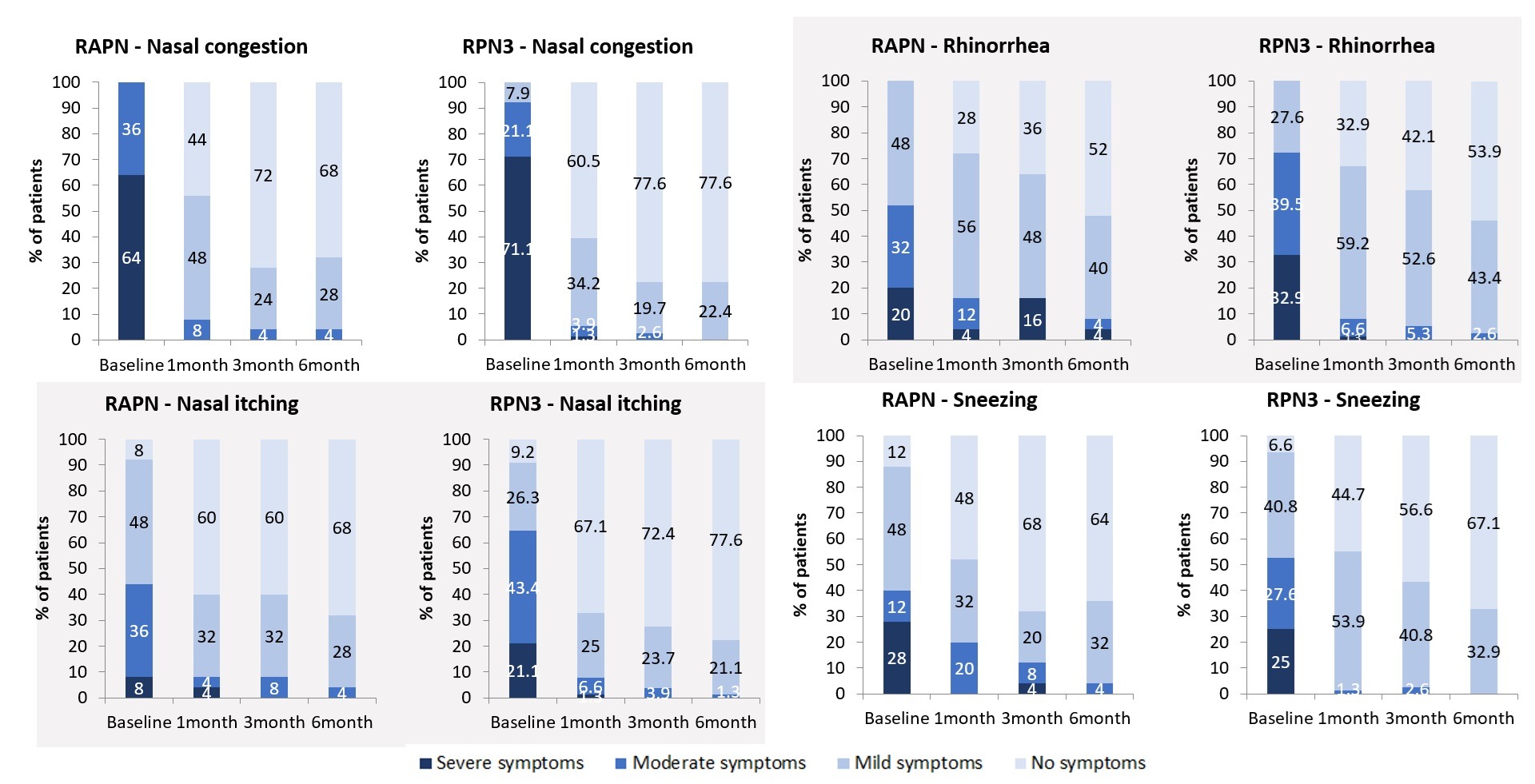

Supplement: Supplementary file 1 — Supplemental Figure. Symptom severity distribution.Severity of rTNSS subscores tracked over time. RPN3 showed greater reductions in rhinorrhea and itching.RAPN, RPN3 as defined above. [file OHN-174-954-s001.jpg]
